# Items

**Published:** 1868-07-01

**Authors:** 


### *Cause of Scurvy.*

Recent experiments upon frogs, dogs, and other animals, by M. Prussak, of St. Petersburg, tend to sustain the older view that scurvy is produced by an excess of common salt in the blood, occasioned by an exclusive salt meat diet. Injections of salt water beneath the skin of frogs, caused the blood corpuscles to distend the vessels, and gave rise to patches of dark colored extravasations, very like scorbutic blotches. It is suggested, in explanation, that excessive osmosis occurs in consequence of the increased density of the blood.

### *New Remedy in Intermittent Fever.*

A correspondent of the *Southern Journal of Medical Science*, says that he has been successful in curing several cases of obstinate intermittent fever, where *Quinine* had failed, by using the *Liquor Ferri Persulphatis.* He usually premised a full dose of *Pil. cath. co.*, and then gave the *Liq. fer. p.* in doses of from eight to fifteen drops every four or six hours.

